# The transcription factor ELF4 alleviates inflammatory bowel disease by activating IL1RN transcription, suppressing inflammatory TH17 cell activity, and inducing macrophage M2 polarization

**DOI:** 10.3389/fimmu.2023.1270411

**Published:** 2023-11-06

**Authors:** Meiwan Cao, Peiyu Chen, Baoling Peng, Yang Cheng, Jing Xie, Ziang Hou, Huan Chen, Liping Ye, Huiwen Li, Hongli Wang, Lu Ren, Liya Xiong, Lanlan Geng, Sitang Gong

**Affiliations:** ^1^ Department of Gastroenterology, Guangzhou Women and Children’s Medical Center, Guangzhou Medical University, Guangzhou, China; ^2^ Center for Child Health and Mental Health, Shenzhen Childen’s Hospital, Shenzhen, China; ^3^ Department of Internal, Guangzhou Women and Children’s Medical Center, Guangzhou Medical University, Guangzhou, China

**Keywords:** transcription regulator ELF4, inflammatory bowel disease, IL1RN, Th17 cells, macrophage M2 polarization, inflammatory factors, disease activity index, bioinformatics analysis

## Abstract

**Background:**

Inflammatory bowel disease (IBD) is a chronic immune-mediated disorder affecting millions worldwide. Due to the complexity of its pathogenesis, the treatment options for IBD are limited. This study focuses on ELF4, a member of the ETS transcription factor family, as a target to elucidate its role in IBD and investigate its mechanism of action in alleviating IBD symptoms by activating IL1RN transcription to suppress the activity of inflammatory TH17 cells.

**Methods:**

Using the GEO database, this study examined LPS-induced intestinal inflammatory genes and their regulation mechanisms. We examined the colon length of LPS-treated mice and derived the Disease Activity Index (DAI). H&E staining, ELISA, and flow cytometry were used to detect mice colon tissue damage, inflammatory factor levels in mouse serum, mouse macrophage types and inflammatory TH17 cell activity. RT-qPCR and Western blot detected ELF4, IL1RN, M1, and M2 polarization markers. *In Vitro*, using dual-luciferase and ChIP assays, we tested mouse bone marrow-derived macrophages (BMDMs) and mouse intestinal epithelial cells for IL1RN promoter activity and ELF4 enrichment.

**Results:**

Bioinformatics showed that LPS-induced colitis animals have reduced ELF4 expression in their colon tissue. *In vivo* tests confirmed reduced ELF4 expression in mice with LPS-induced colitis. ELF4 overexpression reduced mouse intestinal inflammation. ELF4 activated IL1RN transcription in bioinformatics and *in vitro* tests. ELF4 promoted IL1RN transcription and macrophage M2 polarization to limit intestinal epithelial cell death and inflammation and reduce mouse intestinal inflammation *in vitro*. ELF4 also reduced the Th17/Treg ratio by increasing IL1RN transcription.

**Conclusion:**

ELF4 activates IL1RN transcription, suppresses inflammatory TH17 cells, and induces macrophage M2 polarization to treat IBD.

## Introduction

Inflammatory bowel disease (IBD) is a chronic immune disease characterized by recurrent diarrhea, abdominal pain, rectal bleeding, and weight loss. It is mainly divided into Ulcerative Colitis (UC) and Crohn’s Disease (CD) (1). IBD is most common in Western countries, mainly Northern Europe and North America, affecting approximately 1.6 million Americans (2). However, in the past decade, the incidence of IBD has been on the rise among Asians and Hispanics (2). This disease is more common in adolescents and adults aged 15 to 35, with no specific gender preference (3). Studies have shown that the occurrence of IBD results from the interaction between susceptible genes, the environment, and the immune system. The weakening of the intestinal barrier causes intestinal immune overactivation. Among all the pathogenesis mechanisms, immune dysfunction receives the most attention (4).

ELF4 (E74-like factor 4) is an ETS transcription factor family member. It is one of the crucial genes in tumor occurrence, development, and immune system function (5). The initial discovery of ELF4 was in the bone marrow and T-cell lineages, where it activated immune-related molecules such as Interleukin (IL)-3 and Granulocyte-Macrophage Colony-Stimulating Factor (GM-CSF) (6). A recent study reported five cases presenting with oral ulcers, symptoms of inflammatory bowel disease, unexplained fever, anemia, or systemic lupus erythematosus. Through whole exome sequencing, potential ELF4 mutations were identified in all cases (7). The research results of Tyler et al. showed that functional defects of ELF4 were related to high levels of mucosal inflammation and inflammatory bowel disease (IBD). They pointed out that in murine macrophages, Elf4 not only maintained the expression of anti-inflammatory genes such as IL1Rn but also restricted the upregulation of inflammatory factors such as S100A8, Lcn2, Trem1, and neutrophil chemotactic factors, thus inhibiting inflammation (8). These reports have provided us with new ideas for studying the pathogenesis of inflammatory bowel disease.

TH17 cells mainly secrete IL-17A and IL-17F. The latter could bind to cell surface receptors IL-17RA and IL-17RC, activate signaling pathways such as NF-κB, C/EBP, and MAPK, promote the release of inflammatory factors, and enhance mucosal immune response (9). Meanwhile, IL-17A could synergize with TNF-α to enhance the inflammatory response of IBD (10). Macrophages are innate immune cells that can be divided into M1 and M2 according to their activation states (11). During the development stage of IBD, M1 macrophages play a crucial role in phagocytosing pathogens and microorganisms, which is vital for inflammation suppression. M2 macrophages can release anti-inflammatory cytokines and participate in tissue repair after injury, which could alleviate intestinal damage (12).

Despite numerous research reports on IBD in recent years, the treatment methods for IBD still need to be improved due to its complex pathogenesis. This study aimed to clarify the role of ELF4, a member of the ETS transcription factor family, in IBD and explore the mechanism by which ELF4 relieves IBD symptoms by activating the transcription of IL1RN and suppressing the activity of inflammatory TH17 cells. This study provides new targets for IBD research and lays a theoretical foundation for analyzing the pathological mechanisms of IBD. In addition, this study provides a new approach for the clinical application of small-molecule drugs targeting ELF4 to treat inflammatory bowel diseases.

## Materials and methods

### Bioinformatics analysis

The Gene Expression Omnibus (GEO) database (https://www.ncbi.nlm.nih.gov/gds) was utilized to obtain two datasets: GSE97371 and GSE38713. GSE97371 consists of three samples of mouse small intestine tissue treated with LPS and three control samples of small intestine tissue. GSE38713 includes 30 samples of colonic mucosal tissue from patients with ulcerative colitis (UC) and 13 healthy control samples. Differential gene expression analysis was conducted using the “limma” package in R. The criteria for selecting differentially expressed genes were set as |log fold change (FC)| > 2 and P < 0.05. Transcription factors (TFs) were downloaded from the AnimalTFDB3.0 database (http://bioinfo.life.hust.edu.cn/AnimalTFDB/#!/), and the “HP_ABNORMAL_INFLAMMATORY_RESPONSE” gene set was obtained from the MSigDB database (http://www.gsea-msigdb.org/gsea/msigdb/index.jsp). Based on the data from the 30 UC patient colonic mucosal tissue samples in GSE38713, we performed another analysis using the “limma” package in R to identify inflammation-related genes significantly positively correlated with ELF4 expression. The threshold for significance was set as r > 0.5 and P < 0.05.

### Cell culture

DMEM medium (11965092, Gibco, USA) supplemented with 10% fetal bovine serum (FBS, 16140089, Gibco, USA), 100 μg/mL streptomycin, and 100 U/mL penicillin (15640055, Gibco, USA) was used to culture cells from both X Company and Y Company (X Company: 0005, Y Company: Ningbo Mingzhou Biotechnology Co., Ltd., China) in a carbon dioxide incubator set at 37°C. When MODE-K and HEK293T cells reach 80-90% confluence in the culture bottle, trypsin solution (25200072, Gibco, USA) at 0.25% concentration, prepared with 0.5 mM EDTA (Ethylene Diamine Tetraacetic Acid, R1021, Thermo Scientific, USA), was used to digest the cells for a subculture (13).

Bone marrow-derived macrophages (BMDMs) were isolated from the femurs and tibias of C57BL/6 mice. Initially, the bones were flushed with phosphate-buffered saline (PBS) containing 20 U/mL heparin (RP-43138, Invitrogen, USA). Subsequently, a sterile solution of 0.8% ammonium chloride (12125-02-9, Sigma, USA) was used to incubate the bones for 10 minutes to digest red blood cells. Following this, cells were filtered through a 70 µm filter (FSTR070, Beyotime, Shanghai, China) to obtain a single-cell suspension. The cells were then cultured in DMEM medium supplemented with 10% FBS and 10 ng/mL MCSF (HY-P7085, MCE, USA) for 7 days. Throughout the culture, cell morphology and growth were observed daily using an inverted microscope (Nikon Ti-S, Japan). Finally, the isolated macrophages were utilized for subsequent experiments (14, 15).

### Transfection and sorting of cells

The lentiviral vector used in this study was pCDH-CMV-MCS-EF1-Puro (System Biosciences, Mountain View, USA) with a titer of 1x10^9^ TU/mL. To establish stable cell lines, each lentiviral expression vector was co-transfected with a plasmid mixture packaged using Lipofectamine RNAiMAX (13778100, Invitrogen, USA) into HEK293T cells. After 48 hours, the viral-containing supernatant was collected and filtered, followed by infection of BMDM cells in the presence of 8 μg/mL polybrene (428175, Sigma-Aldrich, USA). BMDM cells were cultured for one week in media containing 2 μg/mL puromycin (A1113803, Gibco, USA), and then transferred to puromycin-free media for continued culture to obtain stable transfected BMDM cells. The shRNAs targeting ELF4 and IL1RN were purchased from RiboBio (Guangzhou, China). The sequence for sh-ELF4#1 was 5-CCGCAGGTGTCAACATTCAAGGACACCTT-3, for sh-ELF4#2 it was 5-GACTGTGATTGCTGCCTTTATCAGGACTT-3, for sh-IL1RN#1 it was 5-CCTTTTACCTGAGAAACAACCAGCTCATT-3, for sh-IL1RN#2 it was 5-ATGATATCAAGCTCCAGCTGGAGGAAGTT-3, and the sequence for the non-targeting control sh-NC was 5-CCTAAGGTTAAGTCGCCCTCG-3 (16). Before initial use, perform a brief centrifugation and add RNase-free Water to prepare a storage solution of 20μM.

BMDM cells were divided into the following groups: sh-NC group (infected with sh-NC lentivirus), sh-ELF4#1 group (infected with sh-ELF4#1 lentivirus), sh-ELF4#2 group (infected with sh-ELF4#2 lentivirus), sh-IL1RN#1 group (infected with sh-IL1RN#1 lentivirus), sh-IL1RN#2 group (infected with sh-IL1RN#2 lentivirus), oe-NC group (oe: overexpression, infected with oe-NC lentivirus), oe-ELF4 group (infected with oe-ELF4 lentivirus), oe-NC + sh-NC group (simultaneously infected with oe-NC and sh-NC lentivirus), oe-ELF4 + sh-NC group (simultaneously infected with oe-ELF4 and sh-NC lentivirus), and oe-ELF4 + sh-IL1RN group (simultaneously infected with oe-ELF4 and sh-IL1RN lentivirus). The constructs for oe-NC and oe-ELF4 were designed and synthesized by RiboBio (Guangzhou, China) (14).

TThe co-culture groups of BMDM cells and MODE-K mouse intestinal epithelial cell line were as follows: oe-NC + sh-NC group (BMDM cells co-cultured with MODE-K cells, simultaneously infected with oe-NC and sh-NC lentiviruses), oe-ELF4 + sh-NC group (BMDM cells co-cultured with MODE-K cells, simultaneously infected with oe-ELF4 and sh-NC lentiviruses), and oe-ELF4 + sh-IL1RN group (BMDM cells co-cultured with MODE-K cells, simultaneously infected with oe-ELF4 and sh-IL1RN lentiviruses). Co-culture was performed using the Transwell system, where 1x10^6^ MODE-K cells were seeded in the upper chamber of Transwell, while 1x10^6^ BMDM cells were seeded in a 6-well plate. The Transwell chambers were then placed in the 6-well plate for co-culture. After 48 hours, the Transwell chambers were removed, and the MODE-K cells were collected for further experimental analysis (17).

### Construction of a colitis mouse model

Male C57BL/6 mice (weighing 18~22g, 6 weeks old) were purchased from Shanghai Slik Jingda Experimental Animal Co., Ltd. and housed under constant humidity (45%-50%) and temperature (25~27°C) conditions for 1 week to acclimate to the experimental environment. The mice had ad libitum access to food and water, except for a 12-hour fasting period prior to drug administration. The study procedures followed the guidelines established by the National Institutes of Health and were approved by the Institutional Animal Ethics Committee, ensuring minimal pain, distress, and discomfort to the animals.

The experimental process consisted of a Control group (daily intraperitoneal injection of 0.2 mL PBS for 8 consecutive days) and an LPS group (daily intraperitoneal injection of 0.2 mL PBS for 7 consecutive days; on the 8th day, injection of 0.2 mL PBS solution containing 100 μg/kg LPS). The LPS group was randomly divided into the following groups: LPS + oe-NC (LPS mice infected with oe-NC lentivirus), LPS + oe-ELF4 (LPS mice infected with oe-ELF4 lentivirus), oe-NC + sh-NC (LPS mice simultaneously infected with oe-NC and sh-NC lentivirus), oe-ELF4 + sh-NC (LPS mice simultaneously infected with oe-ELF4 and sh-NC lentivirus), and oe-ELF4 + sh-IL1RN (LPS mice simultaneously infected with oe-ELF4 and sh-IL1RN lentivirus). Each group consisted of six mice. The lentivirus treatment method involved transfecting the respective lentiviral vectors into HEK293T cells and then isolating and purifying the lentivirus. On days 0, 2, and 4 of the LPS treatment, a gastrointestinal injection of the lentivirus was performed, with each injection consisting of 50 μL (1 × 108 TU) (18, 19).

After processing, the mice were euthanized, and their colon tissue was collected. Part of the organization was fixed with 4% paraformaldehyde and then stained, while the other part was stored in liquid nitrogen for subsequent detection by RT-qPCR and Western blot. 10 ng/mL LPS (L2630, Sigma-Aldrich).

### Disease activity index (DAI)

Throughout the experiment, it was necessary to document mouse body weight, fecal characteristics, and fecal occult blood. Scoring was conducted according to the criteria outlined in **Supplementary Table 1** to calculate the Disease Activity Index (DAI): DAI = (weight loss score + fecal characteristics score + fecal occult blood score)/3 (20, 21).

### H&E (hematoxylin-eosin) staining

First, a colon segment approximately 2-3 centimeters in length was excised. Subsequently, it was washed in phosphate-buffered saline (PBS) and the length of the colon was measured from the cecum to the anus. Next, the samples were fixed in 4% paraformaldehyde, embedded in paraffin, and sliced into 5-micrometer sections. These paraffin sections were then subjected to routine dewaxing and graded alcohol hydration. Staining was performed using hematoxylin (IH0030, Beijing Solarbio Technology Co., Ltd., Beijing, China) for 2 minutes, followed by a 10-second rinse with tap water, and then differentiated with 1% hydrochloric acid ethanol for 10 seconds. After a 1-minute distilled water wash, the sections were stained with eosin for 1 minute. Subsequently, the sections were briefly rinsed with distilled water for approximately 10 seconds, dehydrated with graded alcohol and clarified with xylene, and finally sealed with neutral resin. Following the sealing process, the tissue morphology was observed under an optical microscope (XP-330, Shanghai Bingyu Optical Instrument Co., Ltd., Shanghai, China). The corresponding total damage score was determined based on the presence or absence of goblet cell depletion (present=1, absent=0), crypt abscess formation (present=1, absent=0), mucosal structural damage (normal=1, moderate=2, severe=3), degree of muscle thickening (normal=1, moderate=2, extensive=3), and cellular infiltration (normal=1, moderate=2, severe=3) (20).

### ELISA

The levels of inflammatory factors, including Treml, TNF-α, IL-1β, and IL-6, were measured using ELISA kits. This measurement was conducted using either mouse serum or the supernatant of cultured mouse intestinal epithelial cells. For detailed experimental methods, please refer to the instructions provided with the kits (21). To measure the absorbance values, employ an enzyme-linked immunosorbent assay (ELISA) reader at 450 nm, such as the SynergyMx model by BioTek. Analysis can be conducted using Origin 9.5 software.

### Dual-luciferase reporter assay

To assess the activity of the IL1RN promoter, cells were digested and seeded into 35 mm cell culture dishes, which were then placed in a CO2 incubator at 37°C overnight. When the cell density reached 70%, the region sequence of the IL1RN promoter binding ELF4 was cloned into a dual luciferase promoter-reporter plasmid and named pGL3-IL1RN Wild Type (WT) and pGL3-IL1RN Mutant (MUT). ELF4-specific shRNA and oe-NC and oe-ELF4 plasmids were synthesized and designed by Guangzhou Raybio Biotech Company. WT or MUT plasmids were co-transfected with either sh-NC, sh-ELF4, oe-NC, or oe-ELF4 into 293T cells. 48 hours after transfection, cells were lysed and the supernatant collected according to the protocol of the Dual-Luciferase® Reporter Assay System (E1910, Promega). Culture medium was aspirated and cells were washed with pre-cold PBS (without calcium and magnesium ions). 350 μl of pre-cold harvest buffer was added to each dish and the cells were placed on ice for 10 minutes. During this time, sufficient 1.5 mL microcentrifuge tubes were prepared, and ATP buffer and luciferin buffer were mixed at a ratio of 1:3.6 to form the reaction solution, 100 μl per tube. Equal volumes of cell lysate (100 μl) were added to the centrifuge tubes, mixed thoroughly, and the absorbance value was read on a luminometer. The remaining lysate was used to determine the activity, and the readings were used as an internal control to normalize luciferase readings. The ratio of firefly luciferase activity to Renilla luciferase activity (FL/RL) was used as a measure of relative luciferase activity (22). Each experiment was repeated three times.

### Chromatin Immunoprecipitation (ChIP) assay

Cellular DNA-protein crosslinking was induced by treating the cells with 1% formaldehyde and terminated by the addition of glycine solution after 10 minutes. Cells were collected in PBS containing a protease inhibitor, and a sonicator was used to fragment the chromosomes into 200-1000 bp fragments. Each sonication cycle consisted of a 10-second burst followed by a 10-second interval, repeated for a total of 15 cycles. After centrifugation at 4°C, 12000 g for 10 minutes, the supernatant was collected and divided into two tubes. Negative control IgG (ab205718, Abcam, Shanghai, China) and ELF4-specific antibody (1ug, sc-390689, Santa Cruz, USA) were added separately and incubated overnight at 4°C for sufficient binding. The DNA-protein complexes were precipitated using proteinase K/phenol-chloroform and the supernatant was discarded after centrifugation at 12000 g for 5 minutes, followed by washing to remove nonspecific complexes. Subsequently, the crosslinks were reversed overnight at 65°C, and DNA fragments were purified using phenol/chloroform extraction (8, 23). Use qPCR to detect the enrichment of ELF4 on IL1RN promoter, with the primer sequences of IL1RN promoter as follows: F: 5-GAAATCCCCATAGACCCCAAATG-3; R: 5-CGTATGTGGTGTTTTCAGCAAC-3.

### Western blot

The total protein was extracted from the tissue by adding RIPA lysis buffer containing PMSF (P0013B, Beyotime, Shanghai). The protein was then extracted according to the instructions provided with the protein extraction kit (P0028, Beyotime, Shanghai) and the total protein concentration of each sample was determined using the BCA assay kit (P0011, Beyotime, Shanghai). The protein concentration was adjusted to 1 μg/μL, and 100 μL of each sample was boiled for 10 minutes to denature the proteins. The samples were then stored at -80°C for later use.

An 8%-12% SDS gel was prepared based on the target protein band size. Equal amounts of protein samples were loaded onto the gel using a micropipette and subjected to electrophoresis. The proteins were then transferred from the gel to a PVDF membrane (1620177, BIO-RAD, USA). The membrane was blocked with 5% skim milk or 5% BSA at room temperature for 1 hour. The following primary antibodies were added: GAPDH (92310, 1:5000, Cell Signaling Technology, USA), ELF4 (9741, 1:1000, Cell Signaling Technology, USA), IL1RN (NBP1-32568, 1:1000, Novusbio, USA), Arg1 (93688, 1:1000, Cell Signaling Technology, USA), CD163 (68922, 1:1000, Cell Signaling Technology, USA), iNOS (13120, 1:1000, Cell Signaling Technology, USA), Bax (14796, 1:1000, Cell Signaling Technology, USA), cleaved caspase-1 (89332, 1:1000, Cell Signaling Technology, USA), pro caspase-1 (24232, 1:1000, Cell Signaling Technology, USA), Bcl-2 (3498, 1:1000, Cell Signaling Technology, USA), Cleaved Caspase-3 (9661, 1:1000, Cell Signaling Technology, USA), Caspase-3 (9662, 1:1000, Cell Signaling Technology, USA), Cytochrome c (4280, 1:1000, Cell Signaling Technology, USA). The membrane was incubated overnight at 4°C, followed by washing with 1×TBST buffer at room temperature for 3 times, 5 minutes each time.

The membrane was then incubated with a goat anti-rabbit IgG secondary antibody conjugated with HRP (ab6721, 1:5000, Abcam, UK) or a goat anti-mouse IgG secondary antibody conjugated with HRP (ab205719, 1:5000, Abcam, UK) at room temperature for 1 hour, followed by washing with 1×TBST buffer for 3 times, 5 minutes each time. The membrane was immersed in ECL reagent (1705062, Bio-Rad, USA) and exposed at room temperature for 1 minute. The liquid was removed, the membrane was covered with cling film, and the bands were exposed using the Image Quant LAS 4000C gel imaging system (GE, USA). Using GAPDH as the internal reference, the relative expression level of the protein was calculated by the ratio of the intensity of the target band to that of the internal reference band, and the expression levels of each protein were detected (24). Each experiment was repeated three times.

### RT-qPCR

TRIZOL reagent (15596-018, Solarbio, USA) was used to extract total RNA from cell lines and frozen tissue samples according to the manufacturer’s instructions. To measure mRNA expression, total RNA was reverse-transcribed into cDNA using the PrimeScript™ RT-PCR kit (RR014A, TaKaRa, Mountain View, California, USA). Real-time quantitative reactions were performed on the LightCycler 480 system (Roche Diagnostics, California, USA) using the SYBR Premix Ex TaqTM (TaKaRa). Primers were designed and synthesized by Shanghai Universal Biotech Co., Ltd. (**Supplementary Table 2**). β-actin was used as an internal reference. The relative transcription levels of specific genes were calculated using the relative quantification method (2-ΔΔCT method). The 2-ΔΔCt represents the ratio of the target gene expression in the experimental group to the control group, with the formula as follows: ΔΔCT = ΔCtexperimental group - ΔCtcontrol group, where ΔCt = Cttarget gene - Ctinternal reference gene. Ct represents the amplification cycle number at which the real-time fluorescence intensity reaches the set threshold, indicating logarithmic growth of amplification (25–27). Each sample has 3 replicate holes, and the experiment was repeated 3 times.

### Flow cytometry

After collecting the cells of interest, they were fixed in 4% paraformaldehyde at room temperature for 30 minutes, followed by two washes with PBS. Subsequently, cells were washed with 0.3% Triton X-100 for 5 minutes, followed by two more washes with PBS. After thorough washing, cells were blocked with 1% BSA for 1 hour. Next, cells were centrifuged and the supernatant was discarded. Then, 0.25 µg of CD206 labeled with PE (Y17-505, BD Bioscience), 0.25 µg of CD68 labeled with BV421 (566388, BD Bioscience), and 0.25 µg of CD80 labeled with APC (560016, BD Bioscience) were added. After incubating for 30 minutes at 4°C, cells were washed twice with PBS and subsequently analyzed using the BD Accuri C6 flow cytometer (BD Biosciences) (28).

### Statistical analysis

Statistical analysis was performed using R version 4.1.1 (R Foundation for Statistical Computing, Vienna, Austria) and SPSS 21.0 software (IBM, USA). Measurement data was presented as mean ± standard deviation. Independent sample t-test was used for intergroup comparison, one-way analysis of variance was used for multiple group comparison, and Tukey’s *post-hoc* test was used to compare groups. Repeated measures ANOVA was used to compare the data between different time points within each group, and *post-hoc* tests were conducted using Tukey’s method. When P < 0.05, the difference was considered statistically significant.

## Results

### Transcription factor ELF4 was downregulated in the colon tissue of LPS-induced colitis mice

**Figure 1:**
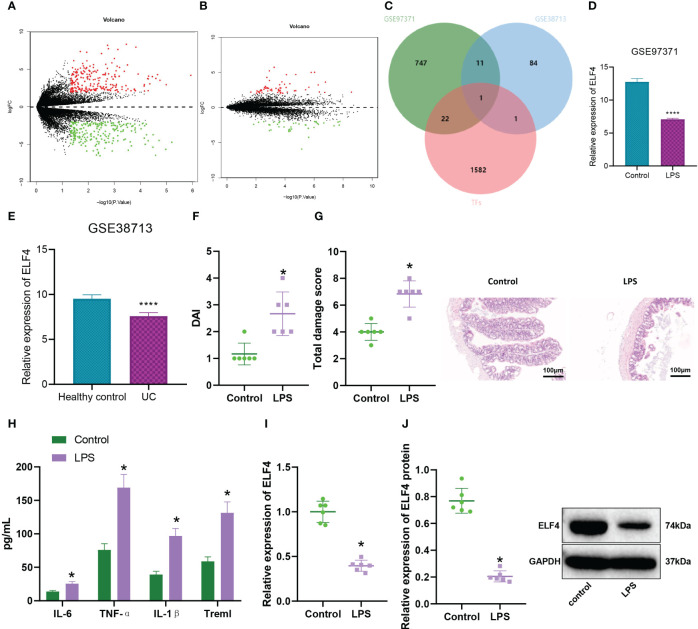
Expression of ELISA in the colon tissue of colitis mice. **(A)** Volcano plot of DEGs between Control group (n=3) and LPS group (n=3) intestinal tissue samples in GSE97371 dataset, with green indicating down-regulated genes and red indicating up-regulated genes; **(B)** Volcano plot of DEGs between Healthy control group (n=13) and Ulcerative Colitis (UC) group (n=30) intestinal mucosal tissue samples in GSE38713 dataset, with green indicating down-regulated genes and red indicating up-regulated genes; **(C)** Venn diagram of the intersection of differential analysis results and transcription factors between GSE97371 and GSE38713 datasets; **(D)** ELF4 expression in Control group (n=3) and LPS group (n=3) intestinal tissue samples based on GSE97371 dataset, where **** represents P < 0.0001; **(E)** ELF4 expression in Healthy control group (n=13) and UC group (n=30) intestinal mucosal tissue samples based on GSE38713 dataset, where **** represents P < 0.0001; **(F)** DAI scores of mice in each group; **(G)** H&E staining of colon tissue damage in mice of each group; **(H)** Levels of inflammatory cytokines IL-6, TNF-α, IL-1β and Treml in serum of mice in each group detected by ELISA; **(I)** mRNA expression of ELF4 in colon tissue of mice in each group detected by RT-qPCR; **(J)** Protein expression of ELF4 in colon tissue of mice in each group detected by Western blot. Each group has 6 samples. * means P < 0.05 compared with the Control group.

Inflammatory bowel disease was a chronic and recurrent intestinal inflammatory disease, mainly including two types: Crohn’s disease (CD) and ulcerative colitis (UC) (29). Studies suggest lipopolysaccharide (LPS) may worsen intestinal inflammation (18). To explore the molecular mechanism of LPS-induced enteritis, we performed differential analysis on the GSE97371 and GSE38713 datasets, selecting the threshold as |logFC|>2 and P<0.05. The GSE97371 dataset contains 3 LPS-treated mouse small intestine tissue samples and 3 control small intestine tissue samples, and the differential analysis results show 781 differentially expressed genes (DEGs) (**Figure 1A**). The GSE38713 dataset contains 30 UC patient colon mucosal tissue samples and 13 healthy control samples. Differential analysis shows 97 differentially expressed genes (DEGs) (**Figure 1B**). Meanwhile, we downloaded 1606 transcription factors (TFs) from the AnimalTFDB3.0 database and compared differentially expressed genes with TFs to obtain a common gene, ELF4, by taking the intersection (**Figure 1C**). Analysis results of dataset GSE97371 showed that compared with the control group, the expression level of ELF4 was down-regulated in the intestinal tissues of LPS-treated mice (**Figure 1D**). The analysis of the GSE38713 dataset revealed that compared to the healthy control group, the expression level of ELF4 was significantly down-regulated in the colonic mucosal tissue of the UC group (**Figure 1E**).

We further induced LPS to establish a mouse model of intestinal inflammation to verify the above results. The results of DAI scoring showed (**Figure 1F**) that compared with the control group, mice in the LPS group had a significant increase in disease activity index. Meanwhile, the H&E staining results (**Figure 1G**) showed that LPS group mice exhibited significant damage to the colonic mucosal tissue, with evident infiltration of inflammatory cells, loss of crypts, mucosal layer disruption, and edema, resulting in a significantly higher total injury score compared to the control group. By ELISA detection of inflammatory factors in mouse serum (**Figure 1H**), we found that Treml, IL-6, TNF-α, and IL-1β levels were significantly elevated in the LPS group mice. In addition, the expression level of ELF4 in colonic mucosal tissues of mice in the LPS group was significantly down-regulated compared with the control group, as detected by RT-qPCR and Western blotting (**Figures 1I, J**).

In conclusion, our study indicates that the expression level of ELF4 was downregulated in the colon tissue of mice with enteritis.

### Overexpression of ELF4 could alleviate inflammatory symptoms in mice’s gut

**Figure 2:**
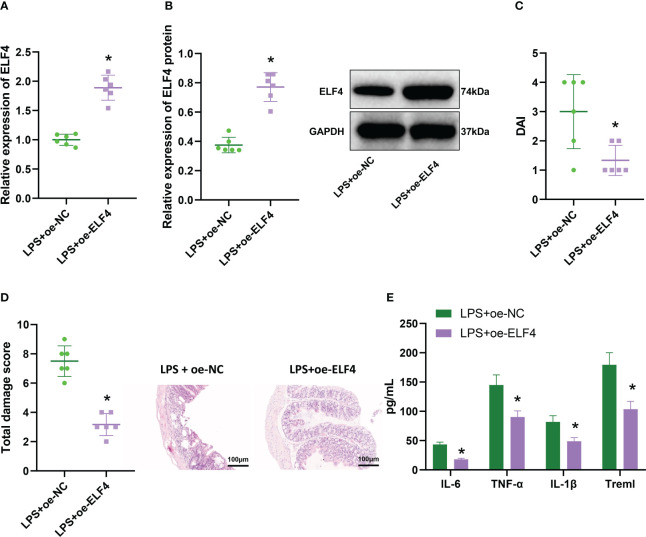
The effect of overexpression of ELF4 on murine intestinal inflammation symptoms. **(A)** The mRNA expression of ELF4 in colon tissues of each group of mice was detected by RT-qPCR; **(B)** The protein expression of ELF4 in colon tissues of each group of mice was detected by Western blot; **(C)** The DAI scores of each group of mice were recorded; **(D)** The colon tissue damage of each group of mice was examined by H&E staining; **(E)** The levels of inflammatory factors IL-6, TNF-α, IL-1β, and Treml in the serum of each group of mice were detected by ELISA. Each group has 6 samples. * means P < 0.05 compared with the Control group.

To investigate the effect of ELF4 on inflammation in the mouse intestine, we used lentivirus overexpressing ELF4 to group mice in our model. Western blot analysis was performed to detect the expression of ELF4; the results showed (**Figures 2A, B**) that the expression of ELF4 in the colonic mucosal tissue of LPS+oe-ELF4 group mice was significantly increased compared with the LPS+oe-NC group. The DAI score results (**Figure 2C**) showed that compared to the LPS+oe-NC group, the DAI of mice in the LPS+oe-ELF4 group was significantly decreased. Meanwhile, the results of H&E staining (**Figure 2D**) showed a significant decrease in inflammatory cell infiltration in the colon mucosa tissue of mice in the LPS+oe-ELF4 group, and the total damage score was significantly reduced compared to the LPS+oe-NC group. ELISA was conducted to measure the levels of inflammatory factors in mouse serum, and the results showed (**Figure 2E**). Compared to the LPS+oe-NC group, the levels of inflammatory factors Treml, IL-6, TNF-α, and IL-1β in mouse serum were significantly decreased in the LPS+oe-ELF4 group. In summary, overexpression of ELF4 could alleviate intestinal inflammation symptoms in mice.

### Activation of the anti-inflammatory gene IL1RN by ELF4 could promote its expression at the transcriptional level

**Figure 3:**
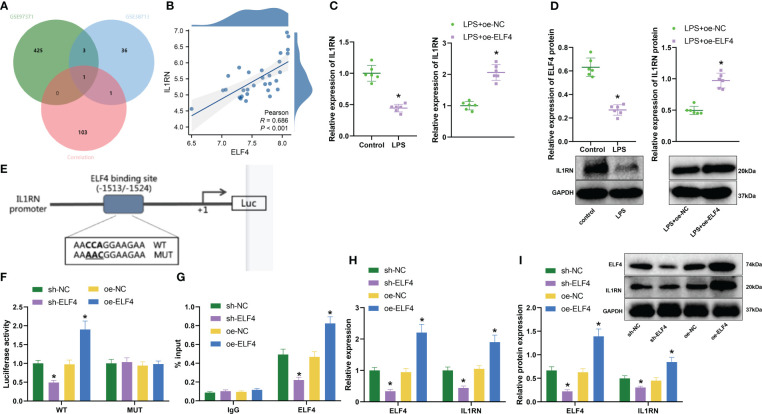
Prediction and validation of downstream target gene IL1RN of ELF4. **(A)** Venn diagram of overlap between downregulated genes and inflammation-related genes in GSE97371 and GSE38713 datasets; **(B)** Correlation analysis of expression between ELF4 and IL1RN (n=30); **(C)** RT-qPCR detection of mRNA expression of ELF4 in colon tissues of mice from each group; **(D)** Western blot detection of protein expression of ELF4 in colon tissues of mice from each group; **(E)** Prediction of ELF4 binding sites on the promoter of IL1RN gene using JASPAR database; **(F)** Dual-luciferase reporter assay to detect IL1RN promoter activity in cells from each group; **(G)** ChIP experiment to detect the enrichment of ELF4 on the IL1RN promoter; **(H)** RT-qPCR detection of mRNA levels of ELF4 and IL1RN in BMDM cells from each group of mice; **(I)** Western blot detection of protein levels of ELF4 and IL1RN in BMDM cells from each group of mice. Each group consisted of 6 samples. * indicates P < 0.05 compared to sh-NC or oe-NC group, ** indicates P < 0.01 compared to Control or LPS + oe-NC group. All cell experiments were repeated 3 times.

Much research indicates that ELF4 primarily functions as a transcriptional activator (30, 31). To identify downstream target genes of transcriptional regulator ELF4, we first selected 429 and 41 significantly downregulated genes based on the GSE97371 and GSE38713 datasets, respectively. In addition, we also downloaded the “HP_ABNORMAL_INFLAMMATORY_RESPONSE” gene set from the MSigDB database, which contains 1164 inflammation-related genes. Then, based on the data of 30 UC patients’ intestinal mucosal tissue samples in the GSE38713 dataset, with a threshold value of r>0.5 and P<0.05, we conducted correlation analysis on ELF4 and 1164 genes and obtained a total of 105 genes significantly positively correlated with ELF4 expression. Next, we took the intersection of 105 genes and the downregulated genes to obtain a common gene, IL1RN (**Figure 3A**). The correlation analysis results show a significant positive correlation between ELF4 and IL1RN expression (**Figure 3B**). Therefore, the transcriptional regulatory factor ELF4 may participate in the anti-inflammatory process by activating the transcription of the anti-inflammatory gene IL1RN.

To verify the above hypothesis, we first used RT-qPCR and Western blot to detect the expression of IL1RN. As shown in the results (**Figures 3C, D**), compared with the Control group, the expression of IL1RN in colonic mucosa tissues of mice was significantly decreased in the LPS group. However, the expression of IL1RN in the colonic mucosa tissues of mice was significantly increased in the LPS+oe-ELF4 group, which was significantly different from the LPS+oe-NC group. In addition, we predicted the binding sites of ELF4 with the IL1RN gene promoter from the JASPAR database (**Figure 3E**) and tested the effect of ELF4 on IL1RN promoter activity using a dual luciferase assay. As shown in the results (**Figure 3F**), the promoter activity of IL1RN was significantly reduced after silencing ELF4, while it was significantly increased after overexpression of ELF4. Notably, we found no effect on the IL1RN promoter activity, whether ELF4 was silent or overexpressed, as long as the binding site of ELF4 was mutated. In addition, ChIP experiment results showed (**Figure 3G**) that silencing ELF4 decreased the enrichment of the ELF4-binding IL1RN promoter, while overexpression of ELF4 significantly increased the enrichment of the ELF4-binding IL1RN promoter. Finally, we also evaluated the levels of ELF4 and IL1RN using RT-qPCR and Western blot. The results showed (**Supplementary Figure 1A**, **Figures 3H, I**) that after silencing ELF4, the levels of both ELF4 and IL1RN were significantly reduced, while after overexpressing ELF4, the levels of both ELF4 and IL1RN were significantly increased. In summary, our experiment demonstrates that ELF4 could enhance the transcription of IL1RN by binding to its promoter.

### *In vitro* cell experiments confirmed that ELF4 could induce M2 polarization of macrophages, suppress apoptosis of mouse intestinal epithelial cells, and generate inflammatory factors by promoting the transcription of IL1RN

According to previous literature reports (8), ELF4 inactivation mutation triggers a high inflammation response, leading to allergic reactions and enteritis in macrophages. Therefore, we hypothesize that ELF4 could enhance the transcription of IL1RN and induce polarization of M2 macrophages, thereby affecting apoptosis and cytokine production in mouse intestinal epithelial cells. We first overexpressed ELF4 or silenced IL1RN in mouse BMDM cells to verify this hypothesis. By detecting through RT-qPCR and Western blot, we found that sh-IL1RN#1 had a better silencing effect. Therefore, we chose it for silencing treatment (sh-IL1RN) (**Supplementary Figure 1B**).

**Figure 4:**
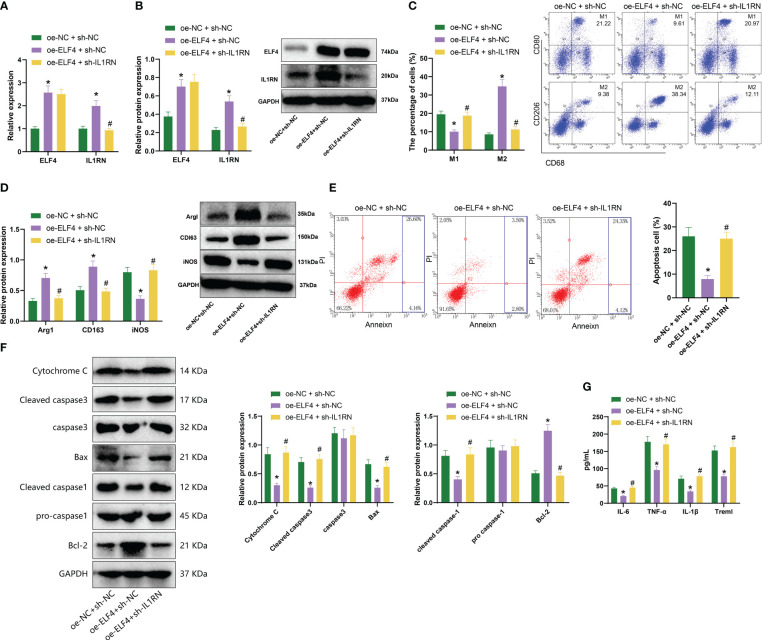
The impact of ELF4/IL1RN axis on macrophages and mouse intestinal epithelial cells. **(A)** The mRNA levels of ELF4 and IL1RN in each group of BMDM cells were detected by RT-qPCR; **(B)** The protein levels of ELF4 and IL1RN in each group of BMDM cells were detected by Western blot; **(C)** The polarization of M1 and M2 in each group of BMDM cells was detected by flow cytometry; **(D)** The protein levels of M1 marker iNOS and M2 markers Arg1 and CD163 in each group of BMDM cells were detected by Western blot; **(E)** The apoptosis of each group of MODE-K cells was detected by flow cytometry (The sum of the blue quadrant areas represents the apoptotic rate); **(F)** The protein levels of apoptosis factors Bcl-2, Bax, cleaved caspase-1, pro caspase-1,caspase 3 and Cytochrome c in each group of MODE-K cells were detected by Western blot; **(G)** The levels of inflammatory factors Treml, IL-6, TNF-α, and IL-1β in each group of MODE-K cells were detected by ELISA. * indicates P < 0.05 compared with oe-NC + sh-NC, and # indicates P < 0.05 compared with oe-ELF4 + sh-NC. All cell experiments were repeated three times.

Next, we designed three experiments: oe-NC + sh-NC, oe-ELF4 + sh-NC, and oe-ELF4 + sh-IL1RN, conducted in mouse BMDM cells. Firstly, we detected the levels of ELF4 and IL1RN by RT-qPCR and Western blot. We found that compared with the oe-NC + sh-NC group, the levels of ELF4 and IL1RN were significantly increased in the oe-ELF4 + sh-NC group, while the level of IL1RN was significantly decreased in the oe-ELF4 + sh-IL1RN group (**Figures 4A, B**). Then, we use flow cytometry to detect the polarization of macrophages. Both M1 and M2 macrophages exhibit the macrophage marker CD68. High CD80 and low CD206 expression characterize M1 macrophages, while M2 macrophages show high CD206 and low CD80 expression (32). Results showed that compared to the oe-NC + sh-NC group, the proportion of M2 macrophages increased while the proportion of M1 macrophages decreased in the oe-ELF4 + sh-NC group. Compared to the oe-ELF4 + sh-NC group, the proportion of M2 macrophages decreased while the proportion of M1 macrophages increased in the oe-ELF4 + sh-IL1RN group (**Figure 4C**). Meanwhile, WB detection of M2 macrophage markers Arg1 and CD163, as well as M1 macrophage marker iNOS, showed that compared with the oe-NC + sh-NC group, the levels of M2 macrophage markers Arg1 and CD163 were elevated while that of M1 macrophage marker iNOS was declined in the oe-ELF4 + sh-NC group; compared with the oe-ELF4 + sh-NC group, the levels of M2 macrophage markers Arg1 and CD163 were reduced while that of M1 macrophage marker iNOS was increased in the oe-ELF4 + sh-IL1RN group (**Figure 4D**).

In further experiments, we co-cultured BMDM cells from each group with the mouse intestinal epithelial cell line MODE-K to assess the apoptosis status of the MODE-K cells. The results showed that compared to the oe-NC + sh-NC group, there was a decrease in the proportion of apoptotic cells in the oe-ELF4 + sh-NC group. Additionally, compared to the oe-ELF4 + sh-NC group, the proportion of apoptotic cells increased in the oe-ELF4 + sh-IL1RN group (**Figure 4E**). Furthermore, levels of apoptosis-related factors Bcl-2, Bax, cleaved caspase-1, and cleaved caspase-3 were measured using Western blotting. The results revealed that, compared to the oe-NC + sh-NC group, the Bcl-2 levels increased in the oe-ELF4 + sh-NC group, while Bax, cleaved caspase-1, cleaved caspase-3, and Cytochrome c levels decreased. The levels of pro-caspase-1 and caspase-3 remained unchanged. Compared to the oe-ELF4 + sh-NC group, the Bcl-2 levels decreased while the Bax, cleaved caspase-1, cleaved caspase-3, and Cytochrome c levels increased in the oe-ELF4 + sh-IL1RN group. The levels of pro-caspase-1 and caspase-3 remained unchanged (**Figure 4F**). Finally, ELISA was used to measure the levels of inflammatory factors in the MODE-K cells. We observed a significant decrease in the levels of inflammatory factors (Treml, IL-6, TNF-α, and IL-1β) in the oe-ELF4 + sh-NC group compared to the oe-NC + sh-NC group. Conversely, the levels of inflammatory factors (Treml, IL-6, TNF-α, and IL-1β) significantly increased in the oe-ELF4 + sh-IL1RN group compared to the oe-ELF4 + sh-NC group (**Figure 4G**).

Therefore, the above results indicate that ELF4 could promote the transcription of IL1RN and induce macrophage M2 polarization, thereby inhibiting apoptosis and inflammation factor production in mouse intestinal epithelial cells.

### Further validation *in vivo* confirmed that ELF4 could alleviate murine intestinal inflammation by promoting the polarization of M2 macrophages by inducing IL1RN

**Figure 5:**
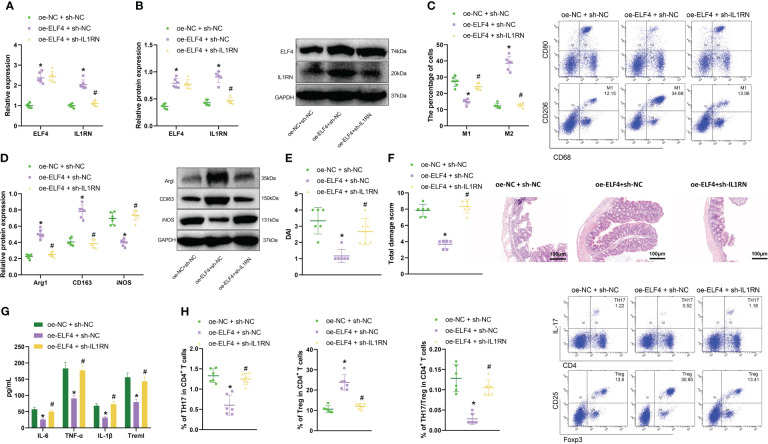
Effects of the ELF4/IL1RN axis on inflammation symptoms in mice intestines. **(A)** RT-qPCR was used to detect the mRNA levels of ELF4 and IL1RN in intestinal tissues of mice from each group; **(B)** Western blot was used to detect the protein levels of ELF4 and IL1RN in intestinal tissues of mice from each group; **(C)** Flow cytometry was used to detect the proportions of M1 and M2 cells in intestinal tissues of mice from each group; **(D)** Western blot was used to detect the protein levels of M1 marker iNOS and M2 markers Arg1 and CD163 in intestinal tissues of mice from each group; **(E)** The DAI scores of mice in each group were recorded; **(F)** H&E staining was used to evaluate colonic tissue injury in mice from each group; **(G)** ELISA was used to measure the levels of inflammatory factors Treml, IL-6, TNF-α, and IL-1β in the serum of mice from each group; **(H)** Flow cytometry was used to detect the proportions of TH17 and Treg cells in intestinal tissues of mice from each group, as well as the TH17/Treg ratio. * indicates P < 0.05 compared with oe-NC + sh-NC, and # indicates P < 0.05 compared with oe-ELF4+sh-NC. All cell experiments were repeated three times.

We further validated the regulatory relationship between ELF4 and IL1RN in a murine model of intestinal inflammation to determine their impact on the disease. We designed three sets of experiments: oe-NC+sh-NC, oe-ELF4+sh-NC, and oe-ELF4+sh-IL1RN. Firstly, we detected the expression levels of ELF4 and IL1RN in the intestinal tissues of each mouse group using RT-qPCR and Western blot. Results showed that, compared with the oe-NC+sh-NC group, the levels of ELF4 and IL1RN were significantly increased in the oe-ELF4+sh-NC group. In addition, compared with the oe-ELF4+sh-NC group, the levels of IL1RN were significantly reduced in the oe-ELF4+sh-IL1RN group (**Figures 5A, B**). Then, we used flow cytometry to detect the polarization of macrophages in the intestinal tissues of each group of mice. The results showed that, compared with the oe-NC+sh-NC group, the percentage of M2 macrophages increased while the percentage of M1 macrophages decreased in the oe-ELF4+sh-NC group.

Compared with the oe-ELF4+sh-NC group, the percentage of M2 macrophages decreased, and the percentage of M1 macrophages increased in the oe-ELF4+sh-IL1RN group (**Figure 5C**). Meanwhile, we also used Western blot to detect the expression of M2 macrophage markers Arg1 and CD163 and M1 macrophage marker iNOS in the intestinal tissues of mice from each group. Results showed that compared with the oe-NC+sh-NC group, M2 macrophage markers Arg1 and CD163 levels were increased, while M1 macrophage marker iNOS levels decreased in the oe-ELF4+sh-NC group. Compared with the oe-ELF4+sh-NC group, levels of M2 macrophage markers Arg1 and CD163 were decreased, while levels of M1 macrophage marker iNOS were increased in the oe-ELF4 +sh-IL1RN group (**Figure 5D**).

Meanwhile, DAI score results (**Figure 5E**) showed that the DAI of mice in the oe-ELF4+sh-NC group was significantly lower compared to the oe-NC+sh-NC group. In contrast, the DAI of mice in the oe-ELF4+sh-IL1RN group was significantly higher than the oe-ELF4+sh-NC group. The H&E staining result also showed (**Figure 5F**) that compared with the oe-NC+sh-NC group, the colonic mucosal tissue lesions were significantly improved in the oe-ELF4+sh-NC group. However, compared with the oe-ELF4+sh-NC group, the colonic mucosal tissue lesions were significantly aggravated in the oe-ELF4+sh-IL1RN group. ELISA test results showed the levels of inflammatory factors in the sera of each group of mice (**Figure 5G**): Compared with oe-NC+sh-NC group, the levels of the inflammatory factors Treml, IL-6, TNF-α, and IL-1β in the sera of mice in oe-ELF4+sh-NC group were significantly decreased. Compared with the oe-ELF4+sh-NC group, the levels of the inflammatory factors Treml, IL-6, TNF-α, and IL-1β in the sera of mice in the oe-ELF4+sh-IL1RN group were significantly increased. In addition, we further used flow cytometry to detect the distribution of inflammatory TH17 cells and anti-inflammatory Treg cells in the intestinal tissues of each group of mice. The results showed that compared with the oe-NC+sh-NC group, the ratio of TH17/Treg cells was decreased in the oe-ELF4+sh-NC group. However, compared with the one-ELF4+sh-NC group, the ratio of TH17/Treg cells was increased in the one-ELF4+sh-IL1RN group (**Figure 5H**). The results above indicate that in mouse intestinal tissue, ELF4 can promote IL1RN transcription, inhibit the activity of inflammatory TH17 cells, induce M2 polarization of macrophages, and alleviate inflammation symptoms in the mouse intestine.

## Discussion

Our research has found that overexpression of ELF4 could alleviate inflammation symptoms in mouse intestines. The pathogenesis of intestinal inflammation is not fully understood, which may result from the combined action of genetic, environmental, immune, and other factors (33). ELF4 belongs to the E74-like factor subfamily of the ETS transcription factor family and regulates immune response and the development of immune-related cells (34). ELF4 is usually expressed in ovaries, placenta, colon, and hematopoietic cells and could inhibit inflammation and prevent mucosal diseases (8). Studies have shown that the absence of ELF4 leads to a decrease in intestinal barrier integrity, known as early-onset mucosal inflammation (35). Mice lacking the Elf4 gene exhibit dysbiosis of intestinal microbiota, characterized by increased Bacteroides (34).

According to our research, ELF4 could promote the transcription of IL1RN to inhibit apoptosis and inflammation factor generation in mouse intestinal epithelial cells. It indicates that ELF4 is a direct positive regulator targeting IL1RN in the mouse intestine. ELF4 promotes the transcription of IL1RN, suppressing apoptosis and inflammatory cytokine production in mouse intestinal epithelial cells. IL-1RN is a member of the IL-1 superfamily, acting as a competitive antagonist of cell-surface IL-1 receptors (36). IL-1RN could affect the sensitivity of IL-1 receptors, inhibiting inflammatory signaling pathways and relieving inflammatory damage to the gastric or duodenal epithelium (37). ELF4 could regulate its inflammatory response, but the specific mechanism by which it suppresses inflammation and prevents mucosal diseases is still unclear. Research has shown that ELF4 is anti-inflammatory and closely related to the IL1RN gene. In mouse macrophages, ELF4 restricts the upregulation of the inflammasome by maintaining the expression of anti-inflammatory genes such as IL1RN (8). Macrophages with ELF4 gene knockout cannot maintain the transcription of the critical anti-inflammatory gene IL1RN but up-regulate pro-inflammatory genes, amplifying the inflammatory response (38).

In addition, we found that ELF4 could suppress the inflammatory activity of TH17 cells and reduce the Th17/Treg ratio by promoting the transcription of IL1RN. Specifically, ELF4 positively promotes the transcription of IL1RN and reduces the activity of TH17 cells by facilitating the transcription of IL1RN. Thus, when TH17 cell activity decreases, the Th17/Treg ratio decreases accordingly. TH17 cells are a subset of Th cells closely related to inflammatory responses, autoimmune diseases, and transplant rejection and have a solid pro-inflammatory effect (39). Treg cells are a subset of Th cells that induce self-immune tolerance and negatively regulate inflammation (40). ELF4 plays a vital role in innate and adaptive immune cells in the immune system. ELF4 deficiency in the embryonic stage may lead to reduced cytotoxicity of NK cells, increased activity of TH17 cells, and abnormal proliferation and transportation of CD8+ T cells (38). In addition, stable-state mice with ELF4 gene deficiency have reduced secretion of IL1RN factor in their blood, increased Th17 cells in their innate layer, and increased Th17 cells in their lymph nodes (8, 41).

Further, *in vivo* animal experiments have confirmed that ELF4 could induce M2 polarization of macrophages by promoting the transcription of IL1RN, thereby relieving intestinal inflammation symptoms in mice. M2 macrophages are generally activated by IL1RN, IL-10, and TGF-β signals. Once activated, they could express high levels of TGF-β, IL-10, M-CSF, vascular endothelial growth factor, etc., thereby reducing inflammation (42, 43). In addition, studies have shown that M2 polarization of macrophages could alleviate LPS-induced intestinal inflammation (44). In mouse macrophages, ELF4 restricts the upregulation of the inflammasome by maintaining the expression of anti-inflammatory genes such as Il1rn, including S100A8, Lcn2, Trem1, and neutrophil chemokines (8). It indicates that M2 polarization of macrophages could be induced by ELF4, thereby alleviating inflammation symptoms in mice intestines.

**Figure 6:**
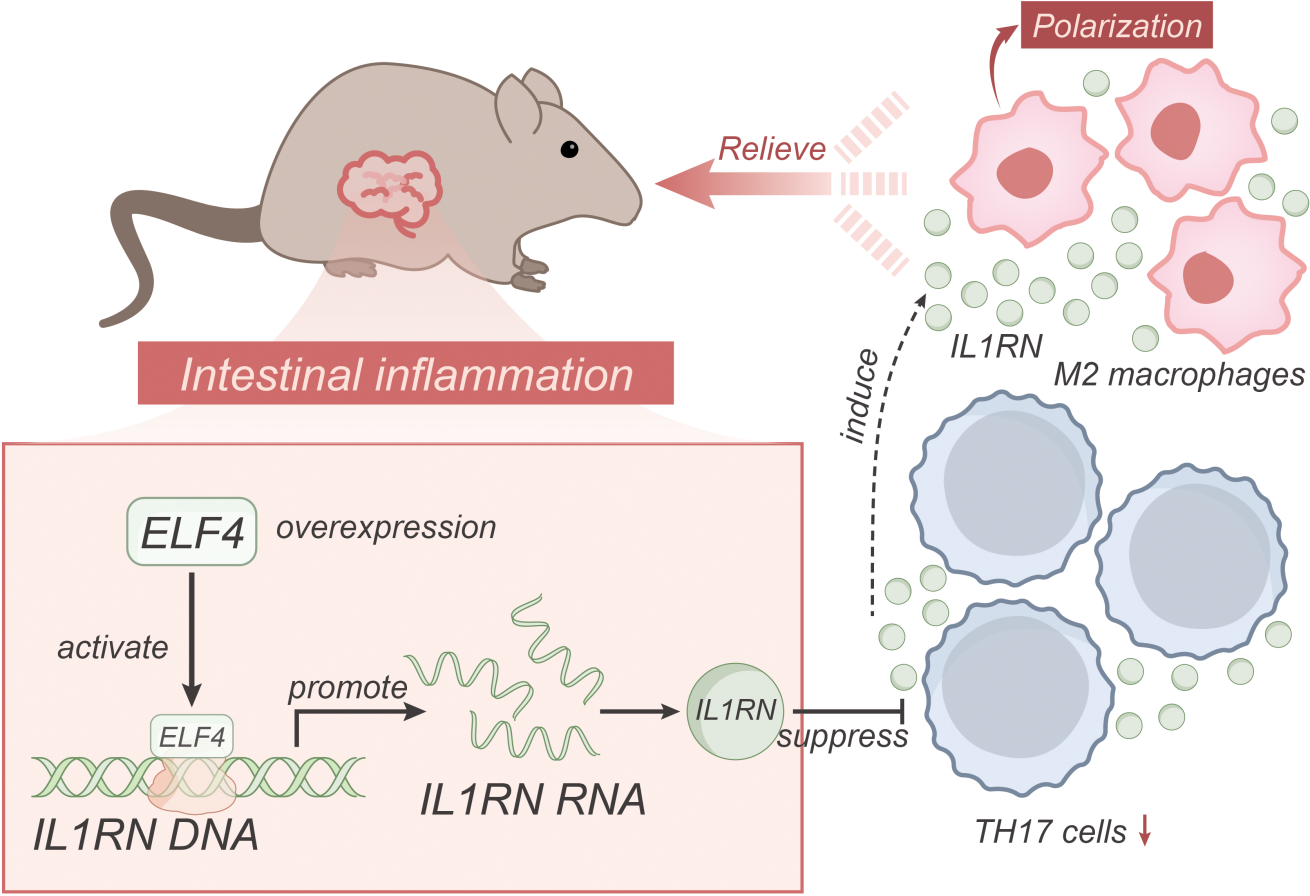
Schematic representation of the molecular mechanism of ELF4, a transcriptional regulator, regulates IL1RN transcription to suppress the pro-inflammatory activity of TH17 cells and induce macrophage M2 polarization in alleviating inflammatory bowel disease.

Based on the above results, we could preliminarily draw the following conclusion: Transcription factor ELF4 may alleviate inflammatory bowel disease by activating IL1RN transcription to suppress the inflammatory activity of TH17 cells and inducing M2 polarization of macrophages (**Figure 6**). The research provides crucial insights into the role of the transcription factor ELF4 in mitigating inflammatory bowel disease (IBD), expanding our understanding of the molecular mechanisms involved in the progression and possible treatments for IBD. By revealing that ELF4 could alleviate intestinal inflammation through activating IL1RN transcription, suppressing inflammatory TH17 cell activity, and inducing macrophage M2 polarization, the study offers a potential therapeutic target for IBD. The clinical value of this research lies in its potential for developing new therapeutic strategies. Given the lack of definitive cures for IBD and the significant impact of this disease on patients’ quality of life, identifying new therapeutic targets like ELF4 and IL1RN offers hope for more effective treatments. The study’s findings may lead to the development of drugs aimed at enhancing ELF4 function or promoting IL1RN transcription, providing a new way to control the progression of IBD and potentially improve the quality of life for patients.

However, the research does have some limitations. First, the study is based on animal models and *in vitro* experiments. While these models could provide essential insights, they may only partially mimic the human physiological environment. Therefore, the applicability of the findings to human IBD needs further confirmation through clinical trials. Secondly, the exact mechanisms underlying the function of ELF4 in activating IL1RN transcription and its subsequent effects remain to be fully elucidated. The study shows that overexpression of ELF4 could alleviate IBD symptoms. Still, it does not explore the possibility of potential off-target effects or the implications of ELF4 overexpression in other biological processes. In future research, the findings from this study should be confirmed in human clinical trials. The role of ELF4 in other inflammatory diseases and the potential side effects of ELF4 overexpression should also be explored to understand its global function in the body better. It would be useful to further investigate the precise mechanisms of ELF4-IL1RN regulation and to look for other possible regulatory factors. Furthermore, developing and testing potential therapeutic agents that could enhance ELF4 function or promote IL1RN transcription would be the logical step in turning these research findings into practical clinical applications.

## Data availability statement

The original contributions presented in the study are included in the article/**Supplementary Material**. Further inquiries can be directed to the corresponding authors.

## Ethics statement

The animal study was approved by the Institutional Animal Ethics Committee of Guangzhou Women and Children’s Medical Center, Guangzhou Medical University (No. 2020-08900). The study was conducted in accordance with the local legislation and institutional requirements.

## Author contributions

MC: Conceptualization, Writing – original draft. PC: Investigation, Writing – original draft. BP: Software, Writing – original draft. YC: Data curation, Writing – original draft. JX: Methodology, Writing – original draft. ZH: Supervision, Writing – original draft. HC: Formal Analysis, Writing – original draft. LY: Project administration, Writing – review & editing. HL: Validation, Writing – review & editing. HW: Resources, Writing – review & editing. LR: Visualization, Writing – review & editing. LX: Investigation, Writing – review & editing. LG: Data curation, Writing – review & editing. SG: Visualization, Writing – review & editing.

## Supplementary material

Supplementary Figure 1 Detection of infection efficiency in BMDM cells. **(A)** RT-qPCR and Western blot were used to detect the silencing efficiency of ELF4 in each group of BMDM cells. #1 had the best effect, so sh-ELF4#1 (sh-ELF4) was used in subsequent experiments. **(B)** RT-qPCR and Western blot were used to detect the silencing efficiency of IL1RN in each group of BMDM cells. * indicates that P < 0.05 compared with sh-NC, and all cell experiments were repeated three times.
