# Supplementary material for: The transcription factor ELF4 alleviates inflammatory bowel disease by activating IL1RN transcription, suppressing inflammatory TH17 cell activity, and inducing macrophage M2 polarization
Source: Front Immunol. 2023 Nov 6;14:1270411. doi: 10.3389/fimmu.2023.1270411 (PMC10657822; doi:10.3389/fimmu.2023.1270411)
Supplement: Supplementary file 1 [file Table_1.docx]

**Table S1. DAI scoring criteria**

| Score | Weight loss(%) | Fecal characteristics | Fecal occult blood |
| --- | --- | --- | --- |
| 0 | 0 | Normal | No latent blood |
| 1 | 1-5% | Mild laxity | Mild bleeding |
| 2 | 5-10% | Relaxation | Moderate bleeding |
| 3 | 10-20% | Mild diarrhea | Severe bleeding |
| 4 | ＞20% | Diarrhea | Hemorrhage |
