# Supplementary material for: The transcription factor ELF4 alleviates inflammatory bowel disease by activating IL1RN transcription, suppressing inflammatory TH17 cell activity, and inducing macrophage M2 polarization
Source: Front Immunol. 2023 Nov 6;14:1270411. doi: 10.3389/fimmu.2023.1270411 (PMC10657822; doi:10.3389/fimmu.2023.1270411)
Supplement: Supplementary file 2 [file Table_2.docx]

**Table S2. RT-qPCR primer sequences**

| Gene | Sequence (5’-3’) |
| --- | --- |
| ELF4-F | ATGCTTGCCAGCCCACTACAGA |
| ELF4-R | CCATTGGTCAGCACCGTAGTCA |
| IL1RN-F | TGTGCCTGTCTTGTGCCAAGTC |
| IL1RN-R | GCCTTTCTCAGAGCGGATGAAG |
| β-actin-F | CATTGCTGACAGGATGCAGAAGG |
| β-actin-R | TGCTGGAAGGTGGACAGTGAGG |

F, forward; R, reverse.
