# Supplementary figures and images for: The transcription factor ELF4 alleviates inflammatory bowel disease by activating IL1RN transcription, suppressing inflammatory TH17 cell activity, and inducing macrophage M2 polarization
Source: Front Immunol. 2023 Nov 6;14:1270411. doi: 10.3389/fimmu.2023.1270411 (PMC10657822; doi:10.3389/fimmu.2023.1270411)

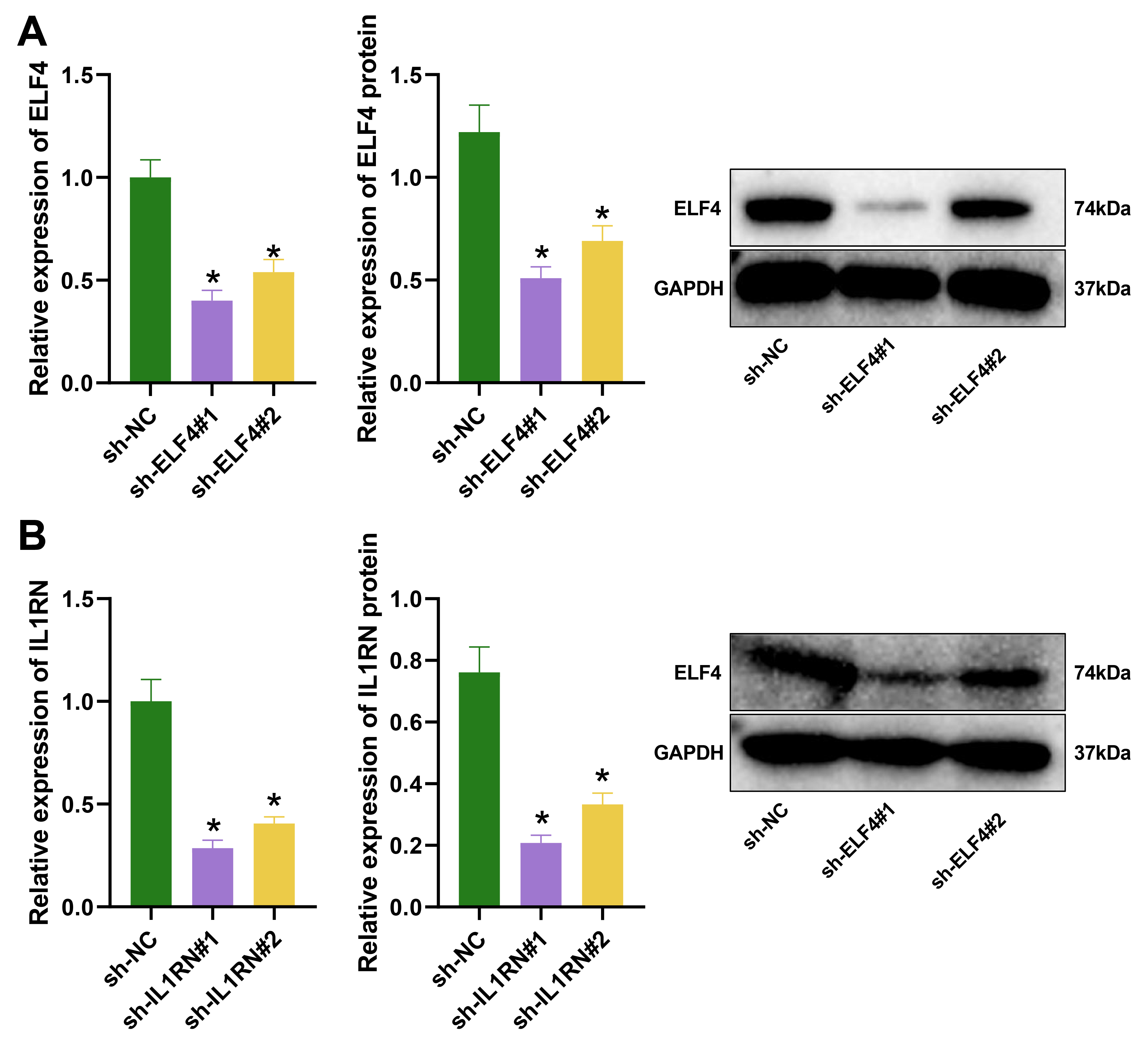

Supplement: Supplementary Figure 1 — Detection of infection efficiency in BMDM cells. (A) RT-qPCR and Western blot were used to detect the silencing efficiency of ELF4 in each group of BMDM cells. #1 had the best effect, so sh-ELF4#1 (sh-ELF4) was used in subsequent experiments. (B) RT-qPCR and Western blot were used to detect the silencing efficiency of IL1RN in each group of BMDM cells. * indicates that P < 0.05 compared with sh-NC, and all cell experiments were repeated three times. [file Image_1.jpeg]
